# Implementation of an ultrasound-guided nerve block program in an academic emergency department

**DOI:** 10.3389/fpubh.2025.1739805

**Published:** 2026-01-27

**Authors:** Joseph Brown, Ryan Tucker, Elizabeth Goldberg, Kelly Bookman, Michael Heffler, Juliana Wilson, Bethany M. Kwan

**Affiliations:** Department of Emergency Medicine, University of Colorado Anschutz, Aurora, CO, United States

**Keywords:** implementation, nerve blocks, regional anesthesia, regional analgesia, point of care ultrasound, POCUS, ultrasound, ultrasound guided nerve blocks

## Abstract

**Introduction:**

Ultrasound-guided nerve blocks (UGNBs) offer effective, opioid-sparing analgesia and have been increasingly recognized as a valuable component of multimodal pain management in the Emergency Department (ED). Despite endorsement by major professional societies, adoption of UGNBs among emergency physicians has been slow due to educational, logistical, and interdepartmental barriers. This study describes the implementation of a comprehensive UGNB program at an academic ED using the Practical, Robust Implementation and Sustainability Model (PRISM).

**Methods:**

A PRISM-guided implementation framework was applied to the University of Colorado Hospital ED (annual census >115,000). Implementation strategies from 2021 to 2025 included hiring a faculty nerve block champion, building interdepartmental collaboration, establishing EHR templates and order panels, creating dedicated nerve block carts and kits, and developing resident training initiatives. Adoption and reach were measured using RE-AIM metrics from registry and electronic health record data.

**Results:**

Zero UGNBs were performed from 2017 to 2020 and only three UGNB were performed in the 6 months prior to program initiation. Following implementation, UGNB procedures increased steadily to over 50 per quarter by mid-2025, encompassing a diverse range of block types. Ninety-three residents and 34 faculty participated in performing or supervising UGNBs. Four ED clinical pathways were updated to include UGNB recommendations, and no patient safety events were reported. Implementation success was facilitated by leadership support, procedural infrastructure, educational programming, and workflow integration.

**Discussion:**

Systematic, framework-driven implementation effectively transformed departmental practice, overcoming long-standing barriers to UGNB adoption. Combining structural changes with educational and cultural interventions fostered sustained procedural uptake and interdisciplinary collaboration. The model presented is reproducible for other EDs aiming to enhance multimodal pain management, reduce opioid use, and institutionalize ultrasound-guided procedures.

## 1 Introduction

Ultrasound-guided nerve blocks (UGNBs) are a core component of a multi-modal approach to managing both acute and chronic pain for Emergency Department (ED) patients. In a UGNB, trained physicians use an ultrasound to identify either a peripheral nerve or a fascial plane through which a nerve (or group of nerves) travel, directly visualize their needle, and accurately deposit anesthetic in the desired area. This targeted deposition of medication under ultrasound guidance improves safety as well as efficacy for the procedure compared to the landmark based approach (1–8). By providing safe and effective analgesia, patients are able to avoid the harmful and potentially dangerous side effects of opiates, which are the most common alternative after other analgesics (non-steroidal anti-inflammatory drugs, acetaminophen, topical patches) have been used (9–13).

Historically, UGNBs have been utilized by anesthesiologists in the peri-operative/operative space due to their dense analgesia/anesthesia. However, in the past two decades, UGNBs have become increasingly leveraged in the ED setting (14, 15). There is a growing body of literature demonstrating both the safety and efficacy of UGNBs when performed by emergency care clinicians (16, 17). UGNBs have been endorsed by the American College of Emergency Physicians (ACEP) as both within the scope of ED-based practice and a critical component of ED-based pain management (18). The American College of Surgeons has also highlighted the use of UGNBs as a core component in the multi-modal approach to traumatic pain (19). Despite this literature and these endorsements, the rate of adoption of UGNB has been slow among emergency physicians (20).

There are many reasons for the slow adoption of UGNB in ED settings (14, 15, 21, 22). From the provider perspective, implementation of UGNBs first requires learning general UGNB principles, specific knowledge of individual block procedures, possible complications, and best practices in medication selection and procedure performance to ensure safety. To augment individual provider knowledge, institutional multidisciplinary safety protocols surrounding medication selection and dosing, as well as duration of observation after UGNBs must be developed. Operational barriers include ensuring sufficient procedural supplies such as nerve block needles, and that appropriate ultrasound machines are available and functional. Finally, intra-departmental collaboration among providers, nursing and medical technicians as well as inter-departmental agreements with services like orthopedics and trauma should be established before initiating a nerve block program to establish best practices around things like pre- and post-procedural neurovascular examinations, frequency of re-checks and dosing of medications (20).

Given the challenge of widescale adoption, this paper outlines the approach of a single academic university hospital ED to implement a UGNB program. A previous effort at our institution identified several barriers to implementation of UGNB for geriatric hip fractures (20). Among the reasons cited for not performing a fascia iliaca nerve block for hip fracture were lack of specific training, discomfort with the procedure, orthopedics consultant objections to performing the procedure, and efficiency concerns. Despite the evidence supporting the use of UGNB for hip fractures, the barriers were so pervasive that in the 18 months prior to intervention, only one UGNB for geriatric hip fracture was performed in our department.

Our objective was to apply the practical, robust implementation and sustainability model (PRISM), a well-established implementation science framework, to describe the resources, infrastructure, processes, and culture change underpinning implementation of this UGNB program. We share strategies and steps involved in leadership engagement, capacity building, and monitoring adoption, integrating the leaders, managers, ED clinicians, and patient perspectives. We report on the uptake of UGNBs over time in response to education and workflow design strategies.

## 2 Materials and methods

### 2.1 Setting and design

This is an implementation science framework-guided evaluation study that demonstrates the process and impact of establishing a UGNB program at the University of Colorado Hospital Emergency Department (UCH ED) in Aurora, Colorado, USA. The UCH ED is an academic ED that sees more than 115,000 patients annually. Of these patient encounters, over 50,000 were associated with pain syndromes based on ICD10 coding (including nearly 20,000 identified as an “injury,” with an additional 30,000 for things like chest pain, abdominal pain or neuropathic pain). In the 3 months prior to intervention, there were three ultrasound-guided nerve blocks (two femoral/fascia iliaca plane blocks and one posterior tibial nerve block) recorded in the UCH ED. In the 47 months prior to that (dating back to July 2017), not a single UGNB was performed in the UCH ED.

### 2.2 Implementation science framework

We applied the practical, robust implementation and sustainability model (PRISM) (23) to guide a post-hoc description of the context and process of implementation of the UCH ED UGNB Program. PRISM includes five contextual domains known to be important for implementation and sustainability of evidence-based interventions such as UGNB. Organizational and patient perspectives on the intervention domains represent factors such as readiness for change, burden, and usability from an organizational perspective and patient-centeredness and access from a patient perspective. Organizational and patient characteristics domains represent factors such as organizational culture, shared goals, clinical leadership support and communication, systems and training, as well as patient demographics, knowledge and beliefs, and disease burden. The implementation and sustainability infrastructure domain represents factors such as a dedicated team, adaptable protocols and procedures, facilitation of shared best practices, and planning for sustainability. The external environment domain represents factors such as reimbursement and regulatory environment. In this report, we focus on organizational characteristics and perspectives on the intervention and implementation and sustainability infrastructure factors.

PRISM also includes RE-AIM (Reach-Effectiveness-Adoption-Implementation-Maintenance), a broadly used implementation outcomes framework (24). This implementation evaluation study is organized according to PRISM contextual domains concerning organizational characteristics and perspectives on the intervention and implementation and sustainability infrastructure, reflecting the experience and perspectives of the UGNB team. We report on Reach (number and characteristics of eligible patients receiving UGNBs), Adoption (number and percent of clinicians being trained and agreeing to deliver UGNBs), and Implementation (frequency of UGNB pathway activation) outcomes as defined by RE-AIM.

### 2.3 Data sources and implementation outcomes

We referenced quality improvement program records to describe the UGNB implementation process and timeline from July 1, 2017 through July 31, 2025. Through group reflection and consensus, the UGNB program leaders, in consultation with an implementation scientist, identified PRISM domain factors pertinent to UGNB implementation and sustainability. We used a combination of data from the NURVE block registry and Epic queries for procedure notes as a standard part of QA/QI to report on nerve block procedure numbers over time to assess RE-AIM Adoption, Reach, and Implementation outcomes. We computed frequencies of UGNB procedures over time.

## 3 Results

### 3.1 Implementation strategies, timeline, and PRISM domains

**Figure 1:**
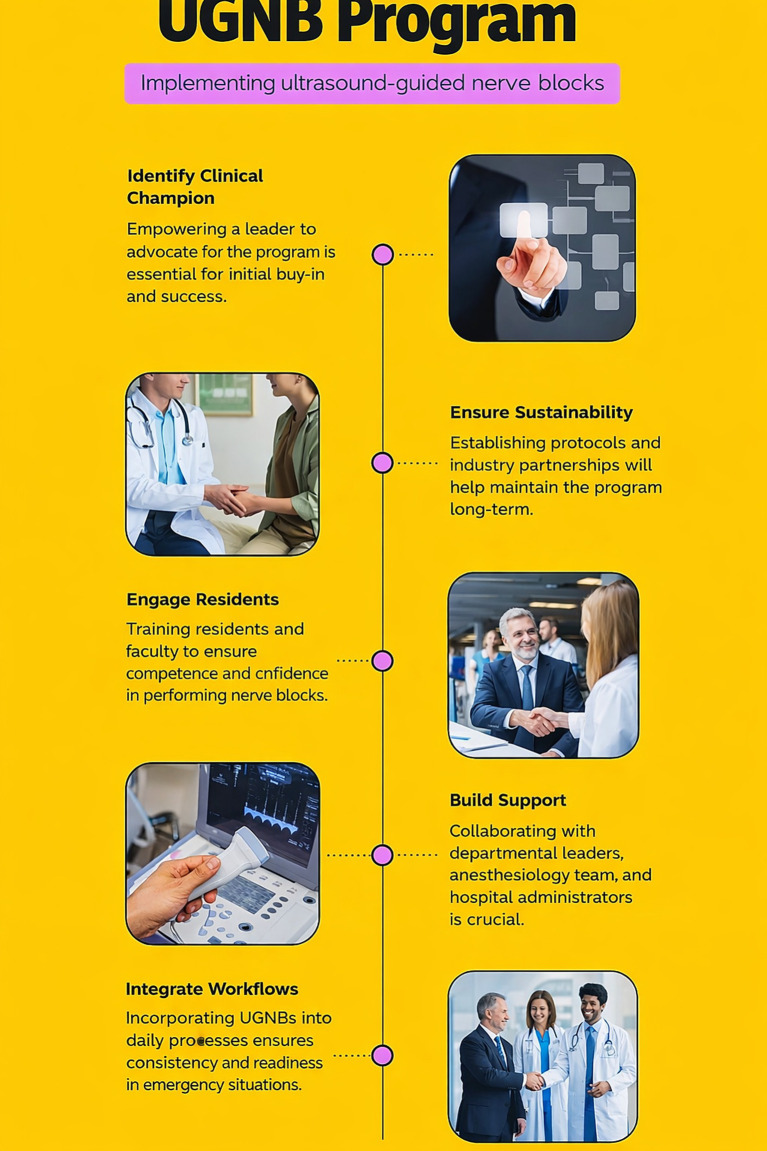
UGNB program implementation flow.

Several strategies were enacted to address the barriers to UGNB adoption and implementation at the organizational and provider level (Figure 1). For each of these strategies, we highlight corresponding organizational-level PRISM domains (organizational characteristics [*PRISM-OC*], organizational perspectives on the intervention [*PRISM-OPI*], implementation and sustainability infrastructure [*PRISM-ISI*]), and alignment with the external environment [*PRISM-EE*] that supported UGNB adoption and implementation (Table 1).

**Table 1:** Ultrasound guided nerve block implementation event timeline.

Date	UGNB program implementation event
September 1, 2021	Intervention STAFF—hiring of Faculty Nerve Block Champions
September 1, 2021–July 2022	Intervention COLLEAGUE level—Discussions with Anesthesia and Acute Pain Service, Orthopedics, and Trauma and Acute Care Surgery to ensure shared model for patient care.
	Intervention SAFETY/STAFF—Discussion with ED Pharmacy—ensure having intralipid (SAFETY) and bupivacaine/ropivacaine (facilitation of flow and operations in ED) in the Pyxis.
	Intervention BILLING—Update Epic Procedure Note to ensure appropriate CPT codes affiliated with correct location and block type.
July 1, 2022	Intervention STAFF—Resident Nerve Block Champion program started. Also did Blocks and Brews (quarterly UGNB educational sessions with residents and DHREM POCUS Fellows)
July 1, 2022	Intervention MATERIALS—UGNB Cart Go Live:
	Stop-Light on Top (COLLEAGUES—agreed upon pathways)
	Johns Hopkins Safe Local App QR code (SAFETY)
	Nerve Block App QR code (STAFF—just in time resource).
	STAFF—Had controlled release syringes, B Braun Stimuplex Nerve Block Needles, Touhy Needles. Pre-printed After Block Instructions.
July 1, 2022	Intervention RESEARCH—updated procedure note for billing and research purposes. One of the founding sites of the NURVE (National Ultrasound-guided neRVE) block registry
August 1, 2023	Intervention STAFF—MH Faculty Hire (EM POCUS Fellowship Trained, Nerve Block Expert)
September 1, 2023	Intervention STAFF—RT Faculty Hire (EM POCUS Fellowship Trained, Nerve Block Expert)
November 2, 2023	Intervention MATERIALS—Departmental deployment of 9 new ultrasound machines. New Ultrasound Machine Deployment with Needle Profiling (SAFETY)
April 10, 2024	ED Clinical Pathways (STAFF) Go Live that were formally agreed upon internally/externally. Geriatric Hip Fracture, Rib Fracture, Pelvic Fracture, Atraumatic Low Back Pain
August 19, 2024	Epic Nerve Block Panel Go Live. Included pre/post nursing monitoring, PRN intralipid (SAFETY)
April 9, 2025	Transition from UGNB Cart to Braun UGNB Kits (SUSTAINABILITY)

#### 3.1.1 Nerve block champion (September 2021)

The initiating event was the hiring of a point of care ultrasound fellowship-trained faculty member with specific expertise in UGNB procedures in September of 2021. This was the first step in establishing a *dedicated team for UGNB [PRISM-ISI]*. Over the following year, this faculty member—henceforth, the nerve block champion (NBC)—was the primary driver of all intra- and inter-departmental collaboration as well as coordinating the materials and electronic health record (EHR) changes required for administering UGNBs. A critical factor in the NBC’s success was consistent *clinical leadership support [PRISM-OC]* for the UGNB program, from leadership’s initial assurances of enthusiasm prior to the NBC’s start date to leadership’s active facilitation of professional connections between the NBC and the ED operations team to support materials construction and necessary EHR modifications, the ED Pharmacy team to ensure quick access to desired medications and intralipid in case of complications, and other relevant consulting services. In addition to these connections, the NBC is afforded time to present program updates at least once annually at the monthly department faculty meetings.

#### 3.1.2 Interdepartmental collaboration (September 2021–July 2022)

**Figure 2:**
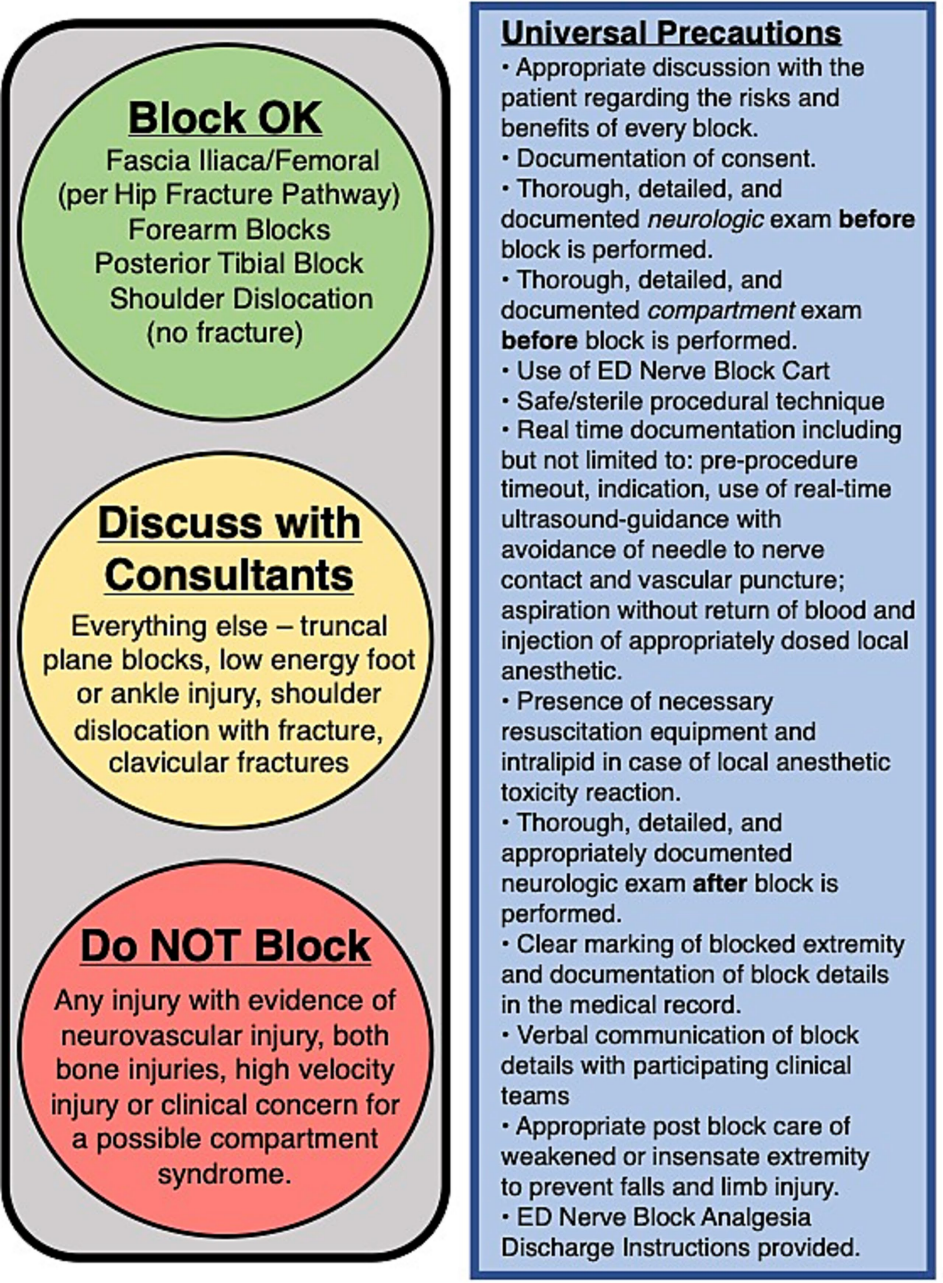
UGNB cart stop light interdepartmental agreement.

The faculty NBC enlisted the support of consulting services that are frequently involved in the treatment of patients who receive nerve block procedures: anesthesia, orthopedic surgery, and trauma surgery. To enhance *coordination across clinical units [PRISM-OPI],* the NBC met with the leadership of these groups to develop a shared model of care that included use of nerve blocks. This was aimed to both perform the procedure earlier in a patient’s stay in the ED and reduce the frequency with which consulting physicians recommended against the use of nerve blocks to ED providers (Figure 2). Additionally, the NBC met with ED nursing leadership to both explain the rationale for UGNBs and the importance of associated nursing orders such as cardiac monitoring and post procedural neurovascular checks which are needed to support patient safety.

**Figure 3:**
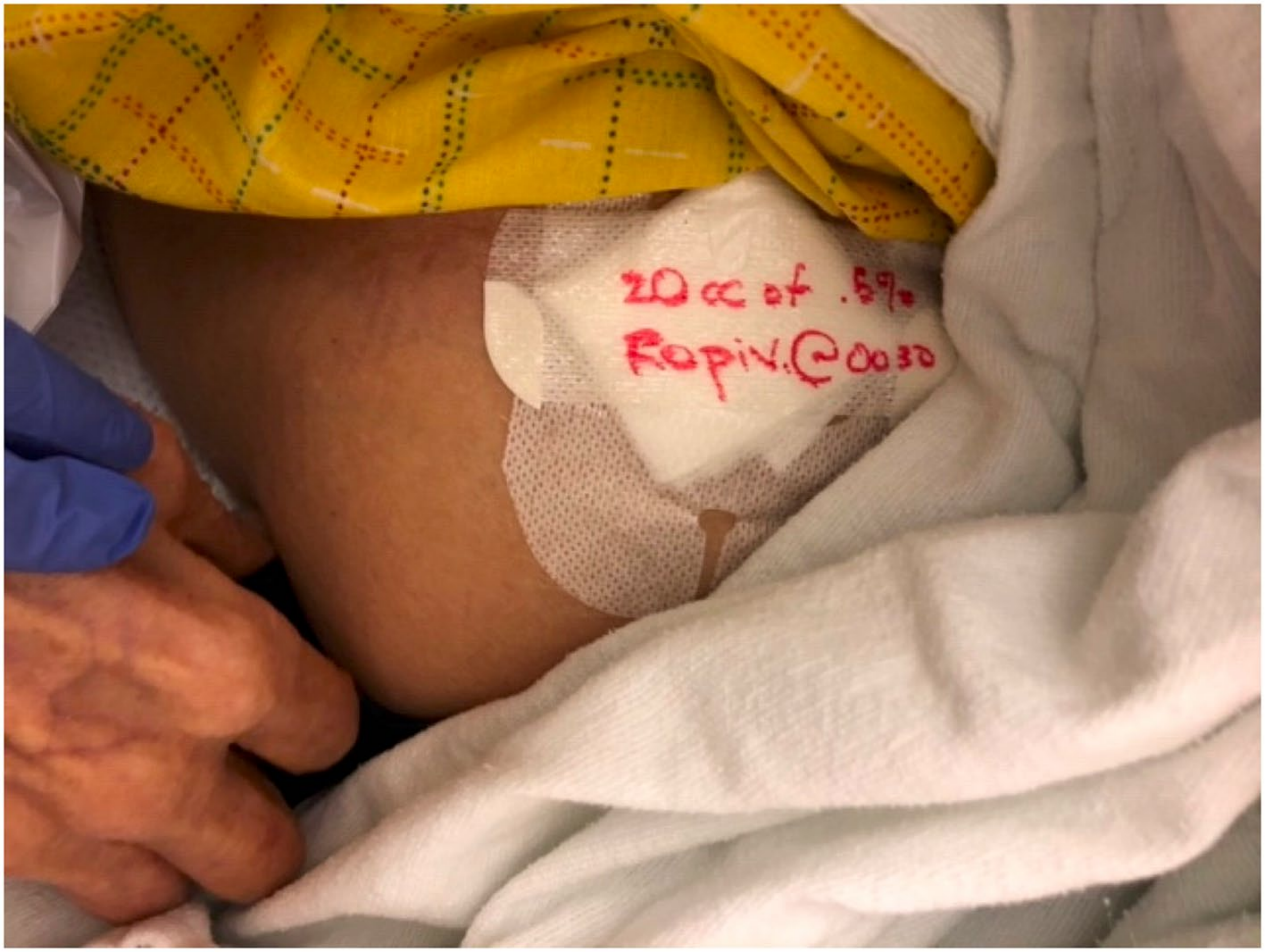
Patient labels after UGNB.

In line with *shared goals and collaboration [PRISM-OC]*, it was confirmed that a shared goal among all parties was to provide safe and efficacious pain control to patients presenting to the ED with acute or chronic painful conditions. As the program has grown there has been increasing acceptance and adoption from the admitting service and at times the ED team is asked to perform the procedure given a multi-year track record of performing UGNB safely and effectively. One of the primary issues raised by anesthesia colleagues was poor real time documentation on UGNBs being done in the ED. As such we established a goal for better on-patient documentation (Figure 3) as well as a push for real time documentation in ED provider notes.

#### 3.1.3 Electronic health record procedure note (September 2021–July 2022)

**Figure 4:**
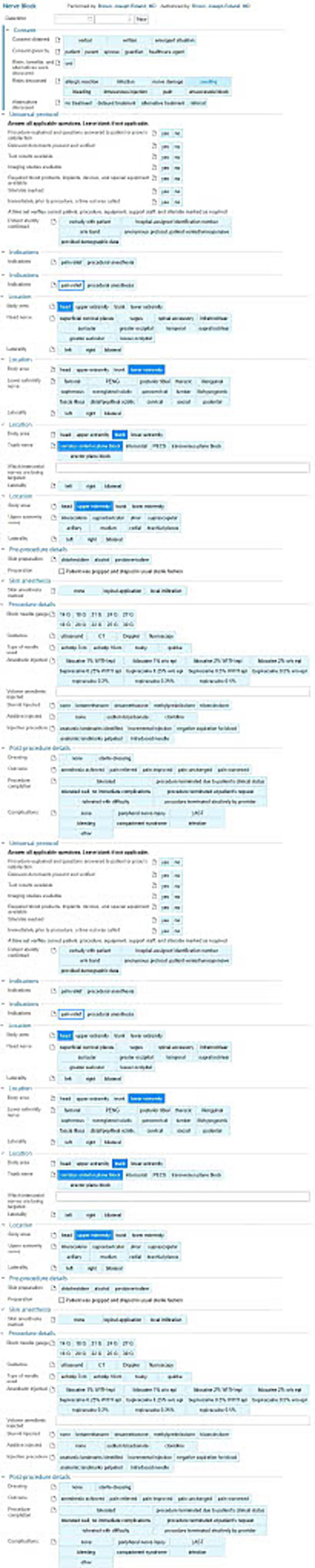
Epic EHR procedure note.

Shortly after starting the program, the nerve block procedure note was updated in the Epic EHR to both ensure adequate billing for the procedures being performed and to push interdepartmental communication (Figure 4). Knowing that revenue generation would be important to support the UGNB program sustainability, the NBC worked with the hospital billing department and Epic analyst team to ensure that the correct Current Procedural Terminology (CPT^Ⓡ^) codes were being generated by the documentation of the nerve block procedures. Another reason for the updated note was the value of research and dissemination of knowledge. The University of Colorado was one of the founding sites of the National Ultrasound-guided neRVE (NURVE) Block Registry, a first of its kind registry of patients receiving UGNB in the ED (16). The new documentation supported information transfer to the registry. The data generated by this procedure note represents *payer satisfaction and reimbursement [PRISM-EE]* factors and provides *performance data [PRISM-ISI]* for monitoring progress, made possible by the department’s informatics resources and commitment to *data and decision support [PRISM-OC]* and aligned with the department’s *expectation of sustainability [PRISM-OC]*.

#### 3.1.4 Resident training program (July 2022)

With these organizational and administrative barriers addressed, the NBC turned his attention to interventions aimed at practicing ED providers in the department—*addressing the barriers of frontline staff, enhancing readiness [PRISM-OPI],* and leveraging organizational *systems and training opportunities [PRISM-OC]*. While there were indications that established faculty in the department were reluctant to integrate UGNBs (20), it was postulated that more junior physicians would not be. Starting in July of 2022, a resident physician NBC (25) effort was instituted—representing *adopter training and support [PRISM-ISI]*. This effort consisted of self-identified resident physicians with a particular interest in nerve blocks and providing them with focused, high yield education so that they were prepared to perform UGNBs in the ED. The first cohort included a total of three residents (one PGY2 and two PGY3 residents). The next group started in July 2023 and had an additional four PGY2 residents. The UGNB program consisted of focused mentorship as well as leading quarterly two-four-hour in-person educational sessions focused on select nerve block procedures. This effort was further supported by both the EM residency program at a local safety net hospital system which has a shared residency program with the UCH ED and the ultrasound fellowship at the same neighboring hospital with quarterly ultrasound sessions in the residency conference—further evidence of *shared goals and cooperation* and *leadership support [PRISM-OC]*.

The faculty NBC met with the resident NBCs quarterly for the first 3 years and would do either individual teaching or answer quality assurance questions. The resident NBCs and Ultrasound Fellows at the neighboring safety net hospital hosted a quarterly evening event for all resident physicians featuring one-two blocks with time to practice scanning—providing an opportunity for *facilitation of sharing best practices [PRISM-ISI]*. Finally, there have been four 90-min ultrasound focused sessions in the resident didactic curriculum annually. Resident and faculty NBCs are also available as an on-shift training resource.

#### 3.1.5 Nerve block cart (July 2022)

**Figure 5:**
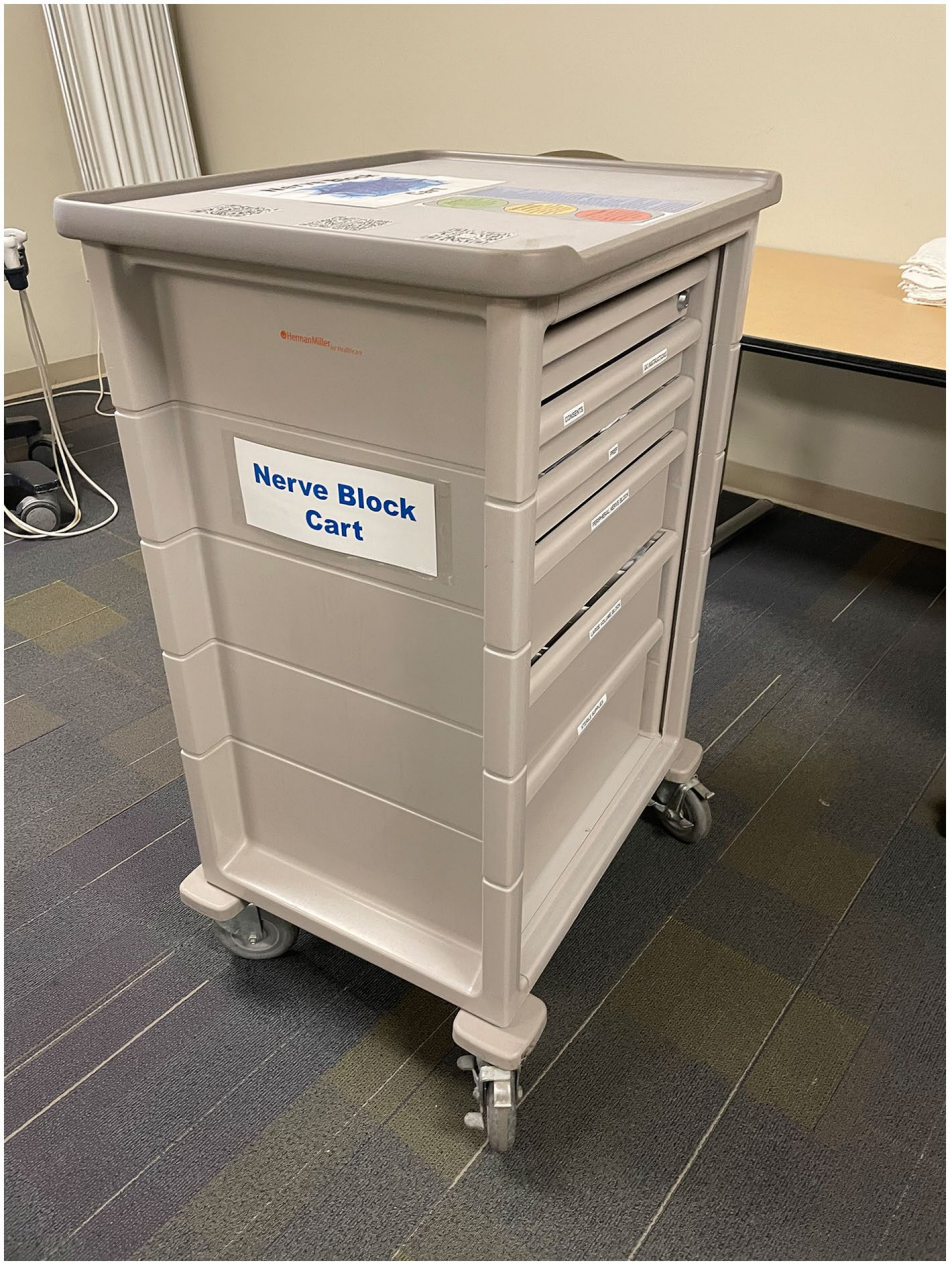
UGNB cart.

From a systems perspective, one of the first steps taken was to ensure safety for patients—a key *barrier for frontline staff [PRISM-OPI]* consideration. Addressing this concern involved internal meetings with both our materials group in the creation of our novel UGNB cart and with ED Pharmacy for medications. A mobile supply cart with all necessary nerve block supplies was created to address concerns about clinical efficiency (Figure 5; Table 2). The cart was stationed in a reliable location in the ED and regularly stocked by the NBCs so that materials were easily accessible. The cart also served to address provider discomfort as it included a QR code to a free iPhone App (26) that serves as a just-in-time resource for reviewing nerve blocks. Additionally, there was a QR code linking to a free iPhone App that facilitates calculating maximum allowable doses of local anesthetic medications to avoid Local Anesthetic Systemic Toxicity (LAST) (27), and pre-printed nerve block aftercare instructions for patients.

**Table 2:** UGNB cart supplies.

Drawer	Item description	Qty
2	CONSENTS	
2	DC INSTRUCTIONS	
3—Prep and Dressing	ALCOHOL WIPES	1
3—Prep and Dressing	CHLOROPREPS	8
3—Prep and Dressing	TEGADERM, 4 × 4	12
3—Prep and Dressing	STERILE GAUZE, 4×4	12
3—Prep and Dressing	STERILE GAUZE, 2×2	12
3—Prep and Dressing	SKIN MARKERS	12
4—Peripheral UGNBs	10CC CONTROL RELEASE SYRINGE	6
4—Peripheral UGNBs	BLUNT FILL NEEDLE	25
4—Peripheral UGNBs	10CC LUER LOCK SYRINGE	10
4—Peripheral UGNBs	22G X 1–1/2” HYPODERMIC NEEDLE	10
4—Peripheral UGNBs	18G X 1–1/2” HYPODERMIC NEEDLE	10
4—Peripheral UGNBs	20G X 3–1/2” TUOHY NEEDLE	6
5—Large Volume UGNBs	20G X 10CM B BRAUN STIMUPLEX NEEDLE	5
5—Large Volume UGNBs	22G X 8CM B BRAUN STIMUPLEX NEEDLE	5
5—Large Volume UGNBs	10CC NORMAL SALINE FLUSH	20
5—Large Volume UGNBs	3-WAY STOPCOCK	10
5—Large Volume UGNBs	EXTENSION TUBING	12
6—Sterile Supplies	STERILE GLOVE-6.0	6
6—Sterile Supplies	STERILE GLOVE-6.5	6
6—Sterile Supplies	STERILE GLOVE-7.0	6
6—Sterile Supplies	STERILE GLOVE-7.5	6
6—Sterile Supplies	STERILE GLOVE-8.0	6
6—Sterile Supplies	STERILE ULTRASOUND PROBE COVERS	12
6—Sterile Supplies	CHUX	2

The cart started with an echogenic tipped needle that was not well visualized under ultrasound, so we quickly pivoted to the B Braun Stimuplex Needle due to its enhanced visualization under ultrasound—demonstrating attention to *usability and adaptability [PRISM-OPI]*. The cart also had preprinted discharge paperwork for patients getting a block then being discharged to facilitate both standardized return precautions and expedite dispositions. For our pharmacy colleagues, we met to ensure that we had intralipid in every Pyxis medication dispenser in the ED in the case of LAST (with no cases of LAST observed in the past 3 years). The cart was built to be entirely comprehensive in that it had all necessary elements for any potential UGNB apart from medications. With time, one of the biggest limitations encountered was the re-stocking of the cart. To better support *sustainability [PRISM-OC]*, the department decommissioned the cart and pivoted to a single use ED Nerve Block Tray in April of 2025 that has been extremely well received and requires minimal manual labor.

#### 3.1.6 Ultrasound-trained faculty (September 2023)

In September of 2023, two additional faculty members were hired and helped support expansion of the UGNB program. Both were fellowship trained in point of care ultrasound (POCUS) and had expertise in nerve blocks (RT, MH). These hires expanded the *dedicated team [PRISM-ISI]* available to support the program.

#### 3.1.7 New ultrasound machines (November 2023)

In the fall of 2023, 9 new ultrasound machines were deployed in the department. These new machines provided improved hardware and imaging quality and introduced new needle profiling technology. This technology affords providers better ability to visualize their needles during UGNB, improving safety of the procedure (28). It is notable that these were purchased because the previous machines were greater than 5 years old and due for updating, not specifically to facilitate UGNBs. O*rganizational leadership support [PRISM-OC]* for improved equipment and amenability to *adaptable protocols and procedures [PRISM-ISI]* enhanced *ability to observe results [PRISM-OPI]* among UGNB providers.

#### 3.1.8 EHR—ultrasound clinical pathways (April 2024)

**Figure 6:**
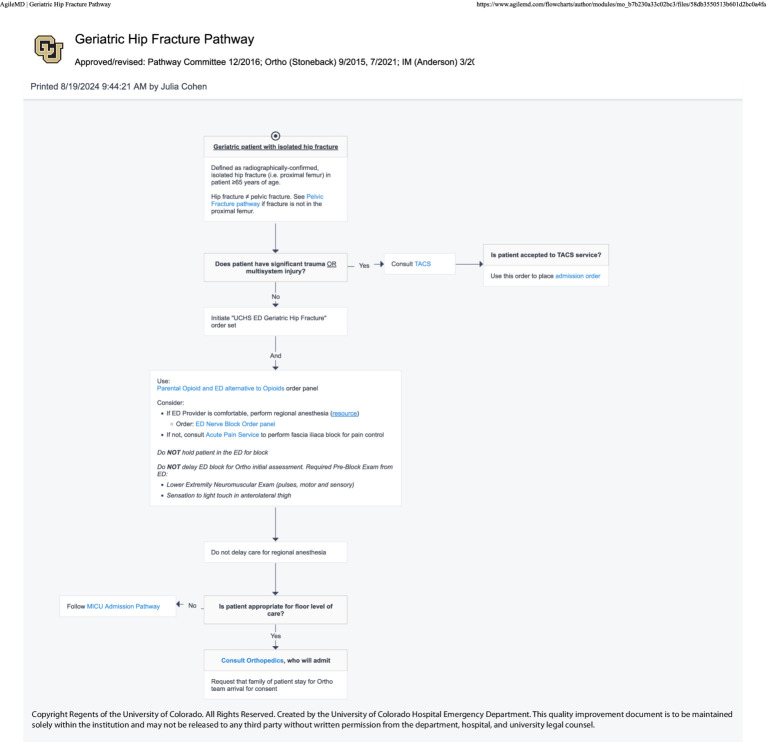
Geriatric hip fracture clinical pathway.

**Figure 7:**
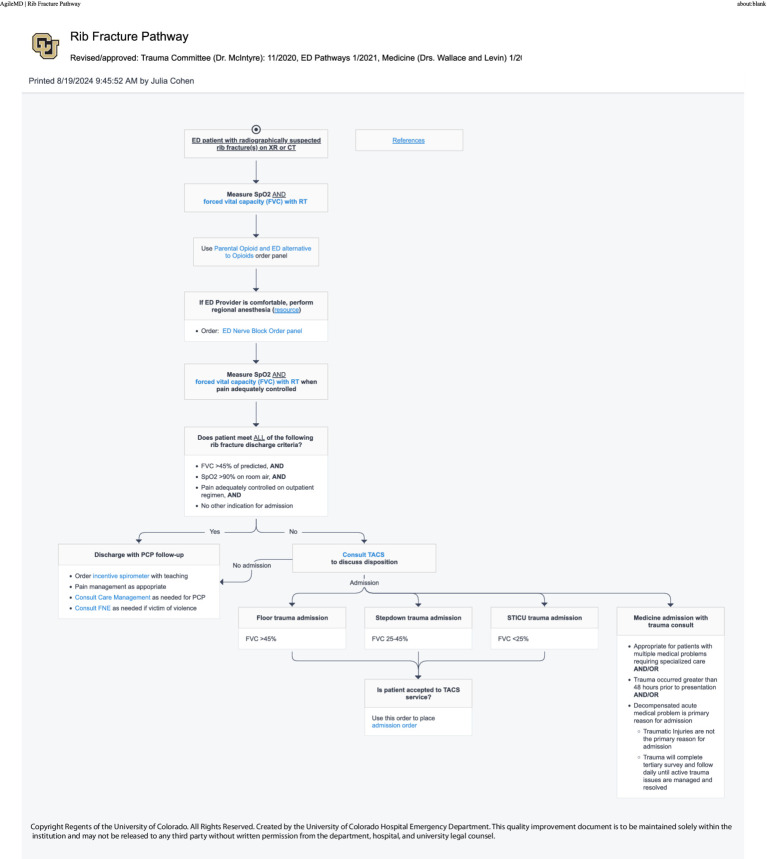
Rib fracture clinical pathway.

**Figure 8:**
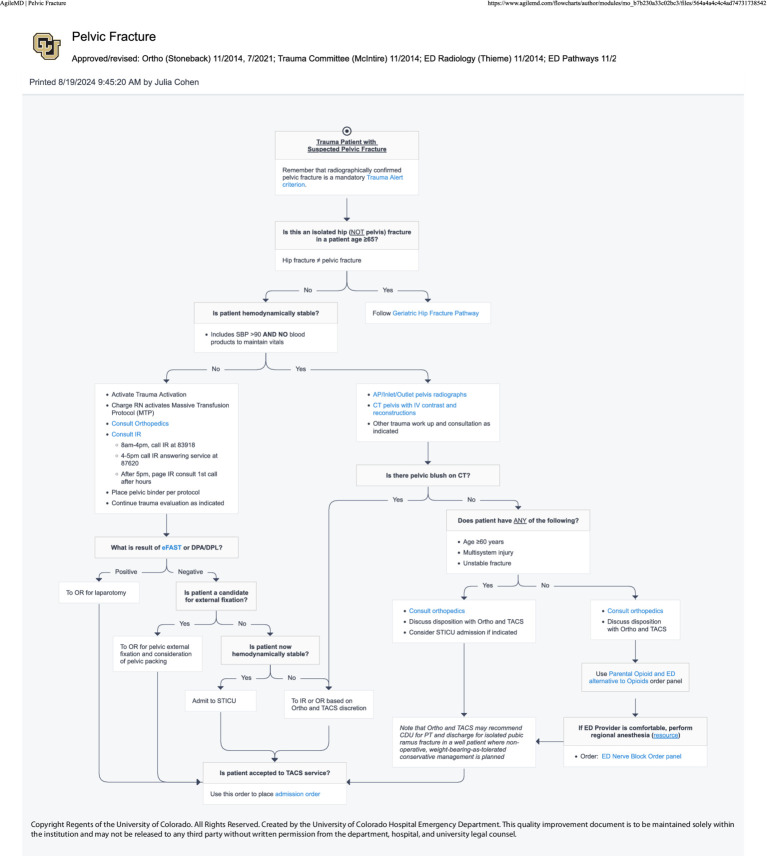
Pelvic fracture clinical pathway.

**Figure 9:**
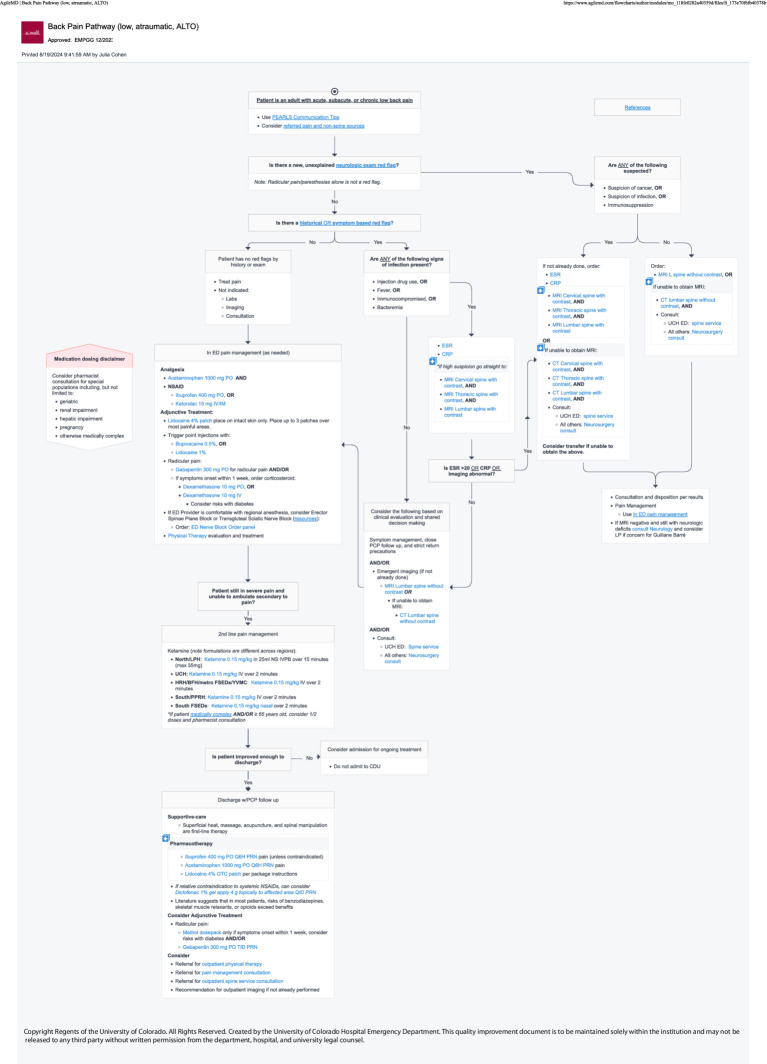
Back pain clinical pathway.

The following spring, the NBC successfully advocated for the addition of nerve block procedures to four key ED treatment protocols (image–clinical pathways). The department is heavily protocol driven, with EHR-embedded, interactive guidelines called “Pathways” existing for commonly encountered scenarios in the ED—an important aspect of *organizational culture and systems [PRISM-OC]*. These pathways are multispecialty, interdepartmental agreements that are consensus driven for evidence-based best practices. Providers in the department are expected to follow these pathways in most cases to provide standardized care to patients. Providers are encouraged to refer to pathways while on shift. However, data surrounding pathway utilization is inherently limited because it requires a provider to access the pathway in order to be tracked and more experienced providers may not use them with every encounter. Patients receiving care via the Geriatric Hip Fracture, Rib Fracture, Pelvic Fracture, or Atraumatic Low Back Pain pathways were identified as most likely to benefit from UGNB (Figures 6–9). Within these pathways, a recommendation to consider an UGNB was added, as well as links to helpful resources such as review of sonoanatomy and procedural details of the relevant UGNB. These changes encouraged providers to perform UGNBs by providing organizational support for the decision as well as reassurance that nerve blocks are safe and effective.

#### 3.1.9 EHR—nerve block order panel (August 2024)

**Figure 10:**
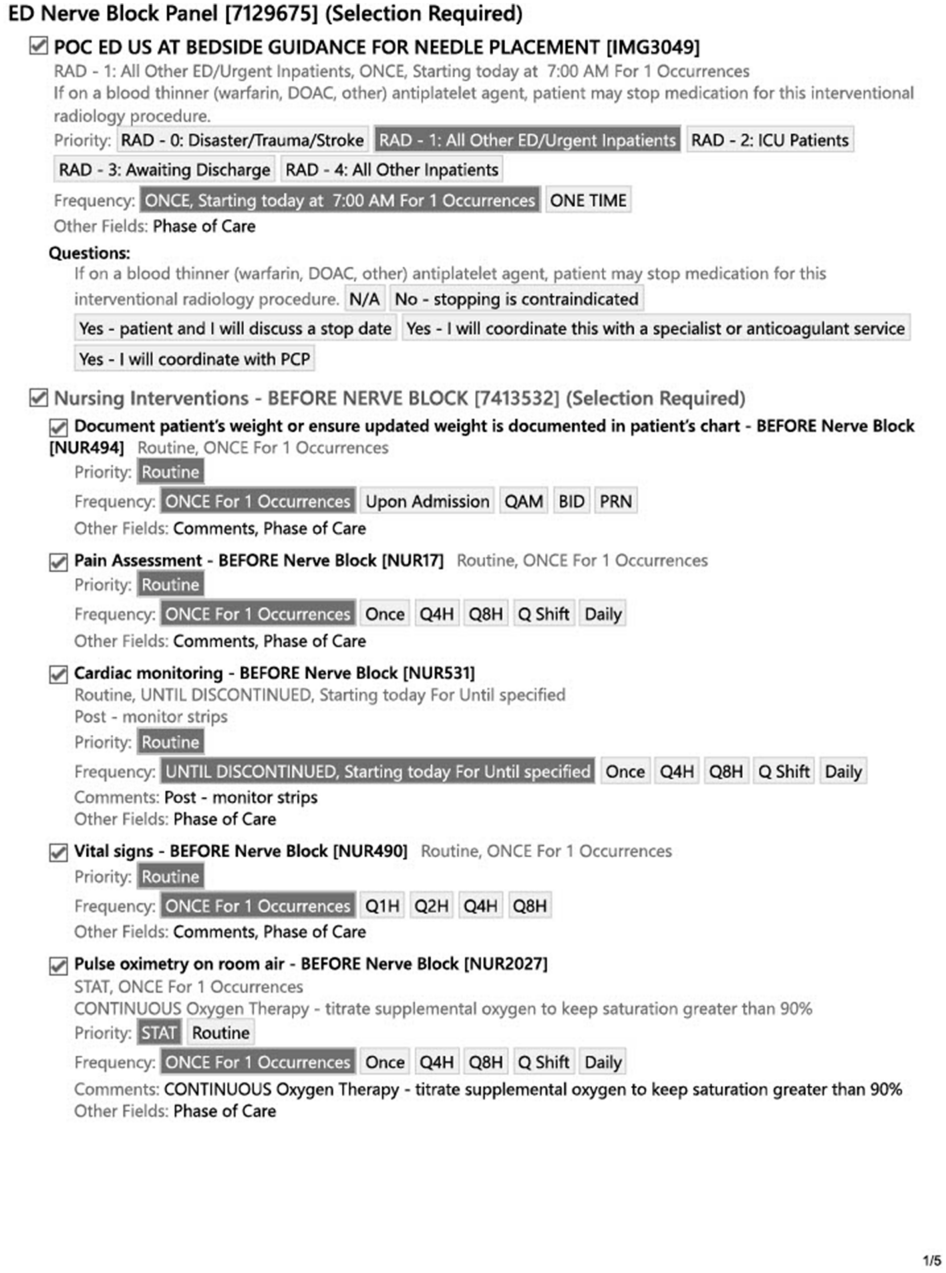
Epic EHR ED nerve block order panel.

To further bolster the safety of nerve block procedures in the department, a nerve block order panel was instituted the following summer. This panel was created in Epic (29) with the help of EHR analysts (Figure 10)—again leveraging department *systems and data infrastructure [PRISM-OC]*. This included easily navigable orders for local anesthetic medications to increase clinician ordering efficiency and weight-based dosing of local anesthetics to reduce the chance of a dosing error and risk of LAST—representing *adaptable protocols [PRISM-ISI]* and *reducing burden and enhancing usability [PRISM-OPI]*. It also included nursing orders for before and after nerve block patient monitoring for patient safety. These orders included placement of cardiac and pulse oximetry monitoring and assessment of pain scores at regular intervals. This would provide feedback to the team about the efficacy of their nerve block in controlling pain as well as creating a protocolized screening process for adverse reactions—a demonstration of the UGNB program’s focus on *relationship and communication with adopters [PRISM-ISI]*. The order panel also included an “as needed” order for intralipid so that it could be accessed quickly in case a patient experienced an adverse reaction to local anesthetic medication. As a part of the new order panel, ED pharmacists would prepare the anesthetic medication(s) in a syringe and hand-deliver it to the ordering provider further supporting safe medication dosing and provider efficiency.

#### 3.1.10 Industry partnership to stock nerve block kits (April 2025)

In the Spring of 2024, nerve block program faculty were made aware by providers that the nerve block cart was not being reliably stocked with necessary materials. Instead of being able to use the cart, clinicians were going to the supply room to find specific materials (such as nerve block needles) in order to perform block procedures. This threatened the efficiency—and thus *sustainability [PRISM-ISI]* of performing the UGNB procedure and potentially undoing the progress that had been made with the initial implementation of the nerve block cart. To address this problem, the NBC worked with a private industry partner BBraun (30) to stock a prefabricated kit specifically tailored to ED nerve blocks. This kit contained all the supplies needed to perform a nerve block procedure with the exception of local anesthetic medications. That April, the nerve block kits were stocked in the supply room, and the nerve block cart was decommissioned—further evidence of *adaptable protocols and procedures [PRISM-ISI]*.

### 3.2 RE-AIM setting- and provider-level adoption and implementation

Since initiation of the UGNB program, 93 different resident clinicians and 34 different faculty have performed or supervised an UGNB *[RE-AIM Adoption]*. The 4 Pathways containing nerve blocks were accessed a total of 2,276 times during the study period *[RE-AIM Implementation]* (Table 3).

**Table 3:** Frequency of pathways triggered.

Pathway title	Pathway opens
Geriatric hip fracture	133
Pelvic fracture	65
Back pain pathway (low, atraumatic, ALTO)	1,509
Rib fracture	569

Adoption at the physician level has been driven primarily by the residents and a few faculty members, including the founding faculty NBC and two additional POCUS-fellowship trained faculty members who have joined the team. Faculty educational sessions were offered shortly after residency conference as well as after faculty meetings in an effort to engage faculty after meetings at which they would already be present. However, these were sparsely attended, with rarely more than one-two faculty members. Subsequently, efforts were dedicated toward residents, with the aforementioned focus during conference as well as evening social events that also covered specific blocks. Resident physicians have been extremely eager to incorporate UGNB into their practice. As such they have done their best to build a culture of collaboration in the ED and in their didactic curriculum. Communication happens via both quality assurance mechanisms as well as via email whenever a new process is being deployed (new clinical pathways, new order panel, transition from cart to trays). Resident physicians are always supervised by a faculty member. Junior residents are typically also supervised by senior residents. When faculty members have been less familiar with a specific UGNB, there has been a culture of support such that trained faculty will leave their zones to offer additional support during the procedure.

### 3.3 RE-AIM patient-level reach

**Figure 11:**
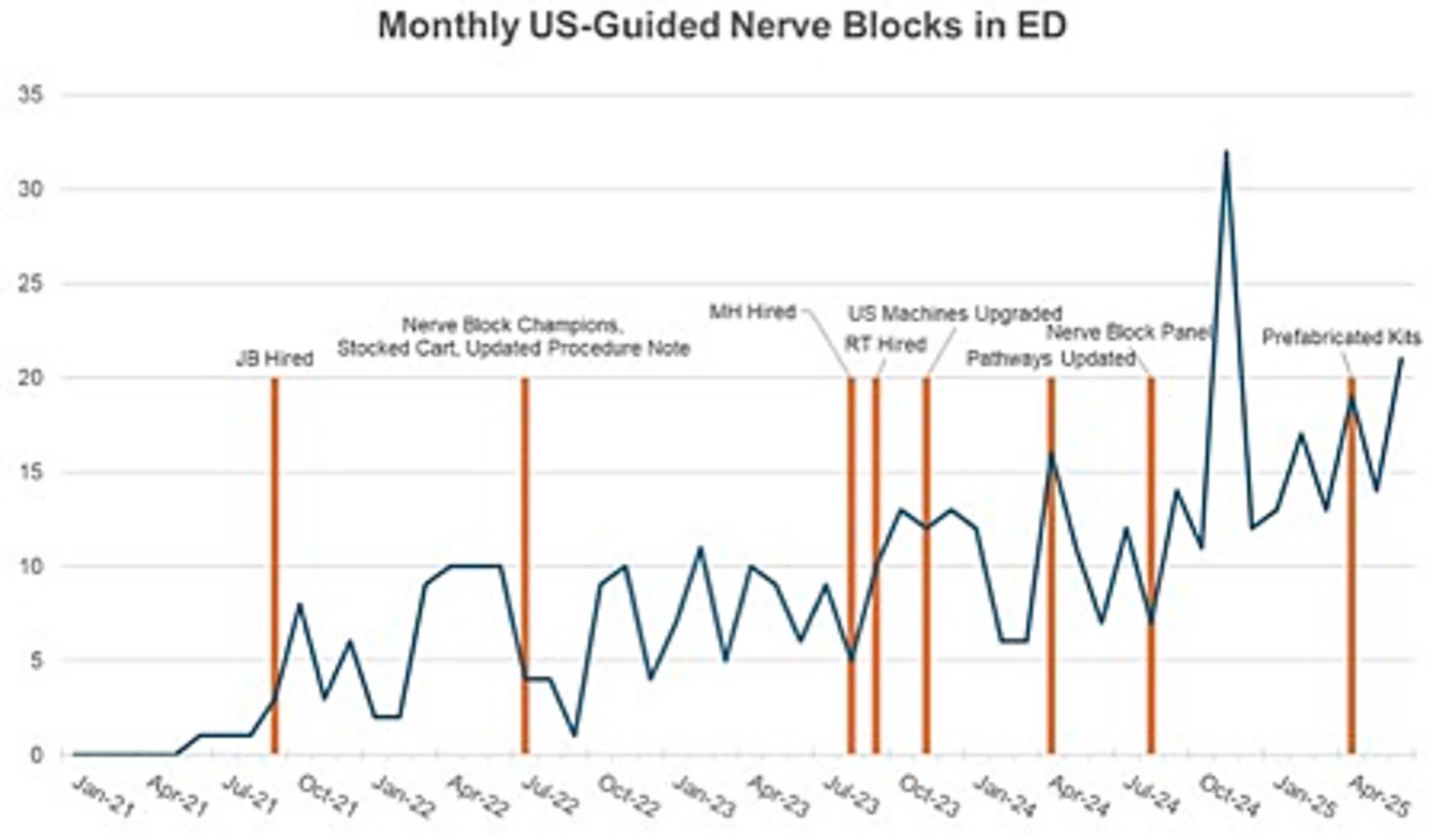
Graph of monthly total UGNB with vertical markers of implementation milestones.

During the nearly 4 years of monitoring post-implementation, the UCH ED went from performing zero UGNBs in the first quarter of 2021 to over 50 in the second quarter of 2025 with a steady, maintained increase every 6–12 months (Figure 11). Additionally, while initial interventions were focused on hip fractures and subsequently rib fractures, there was also an increase in the variety and diversity of types of UGNB performed over time. This included more advanced UGNB like the genicular block for knee pain, a transversus abdominis plane (TAP) block, clavipectoral plane block, dorsal penile block and a stellate ganglion block in a cardiac arrest (Table 4).

**Table 4:** Number of different types of nerve blocks performed in 6-month increments.

Types of blocks	Jan-June 2021	July-Dec 2021	Jan-June 2022	July-Dec 2022	Jan-June 2023	July-Dec 2023	Jan-June 2024	July-Dec 2024	Jan-June 2025
Stellate Ganglion	0	0	0	0	0	0	0	1	0
Supraclavicular	0	0	0	0	0	1	1	1	0
Interscalene	0	0	5	5	6	5	9	6	8
Forearm	0	1	3	2	5	2	1	2	2
Serratus Anterior	0	1	4	3	1	7	6	11	8
Erector Spinae	0	0	10	6	11	14	21	30	30
Fascia Iliaca	1	15	13	14	16	18	13	31	32
PENG (Pericapsular Nerve Group)	0	0	0	0	0	0	1	3	3
Transgluteal Sciatic	0	0	2	2	9	10	2	3	12
Popliteal Sciatic	0	2	3	0	0	1	2	0	1
Posterior Tibial	0	2	1	0	0	1	0	0	0
Superficial Cervical Plexus	0	1	2	0	0	1	2	0	0
Genicular	0	0	0	0	0	1	0	0	0
TAP (Transversus Abdominis Plane)	0	0	0	0	0	1	0	0	0
Clavipectoral	0	0	0	0	0	0	0	0	1
Dorsal penile block	0	0	0	0	0	0	0	0	1
	1	22	43	32	48	62	58	88	97

## 4 Discussion

In this study, we describe the implementation of an UGNB program through incremental improvements in organizational and provider-level support for these procedures, organized according to key implementation and sustainability factors as defined by PRISM. The UGNB program implementation strategies resulted in an exponential increase in completed nerve block procedures in the department over the study period, when zero had been completed in the immediate pre-study period. These findings suggest that a concerted focus and incremental approach to removing organizational and provider-level barriers to nerve block procedures can be effective. A primary driving strategy was hiring a faculty nerve block champion (NBC). When the faculty NBC was hired, he was the primary party interested in increasing the footprint of UGNB in the UCH ED. As such, with critical organizational leadership support and encouragement, he coordinated the intra- and inter-departmental meetings to make this happen. Subsequently, with the recruitment of resident champions there were more clinicians on any given shift looking for an opportunity to do UGNB. Later, there were two additional ED physicians who were both POCUS trained and had expertise with UGNB to help facilitate nerve blocks on shift. It is also worth noting that with each passing year, more new residency graduates were comfortable performing these procedures.

Based on the previous attempt at implementing UGNBs for geriatric hip fractures (20), multiple different approaches were taken to facilitate previously identified barriers. The first barrier was concerns regarding delays in care and disposition. As such, the nerve block cart and then the transition to the single use kits were used to ensure all materials were immediately available. Similarly, all regularly used medications were ensured to be available in ED Pyxis. Finally, the ED Nerve Block Panel was created to ease ordering for clinicians. All updates were made with the frontline provider in mind to limit the additional burden to a clinician. The materials used (nerve block cart and single use kits) were leveraged due to the fact that providers would not have to take multiple steps to perform the procedure. The creation of the panel was to limit the number of clicks a clinician would have to make in the chart and to ensure safety for patients. The update to the procedure note ensured billing capture as well as facilitating research capture. With regards to concerns from consultants, clinical pathways were updated to help ease the decision on when a block was appropriate. Lastly, the ED NBCs were established as a group of clinicians with additional time spent with education and mentoring to help facilitate the performance of UGNB. Based on feedback from consulting providers, the Nerve Block Panel was established to ensure safety when performing UGNB. Additionally, four separate clinical pathways were updated to include UGNB; Geriatric Hip Fractures Pathway uses either Fascia Iliaca or PericCapsular Nerve Group (PENG) block, Rib Fractures Pathway uses either Serratus Anterior Plane or Erector Spinae Plane blocks, Pelvic Fractures uses the PENG block and the Back Pain Pathway uses either the TransGluteal Sciatic Nerve or Erector Spinae Plane block.

Each implementation was made to maximize usability for the practicing clinician. The materials used (nerve block cart and single use kits) were leveraged to facilitate the performance of the UGNB procedure. When something was found to need changing (the limited re-stocking of the nerve block cart), the department was adaptable and changed to the single use kits. Similarly, when the initial nerve block needles were found to not image well on ultrasound, the department supported a change to a different needle. An effort to update both the procedure note and to create the Nerve Block panel was also designed to enhance the performance of the procedure.

One previous study describes the implementation of an ED UGNB program in a community-based ED, in contrast to the academic ED in our study (31). Similar to our study, interdepartmental protocols were developed and implemented. This program also utilized a nerve block cart with necessary supplies similar in composition to ours. As in our study, ED pharmacy was enlisted to ensure supply of anesthetic medications and intralipid. In contrast to our study, the community group had 3 ultrasound fellowship trained faculty with UGNB experience at the time of onset of the program as opposed to one at our institution. This community study also implemented their program at multiple sites including a main ED and 2 associated freestanding EDs. The types of UGNBs performed also differed significantly from those in our department, likely related to differing patient populations. Pre- and post-implementation outcomes were not reported for this previous study.

Our study has several limitations. First, the utilization of UGNB procedures is likely increasing across EDs in the United States as the data supporting its use increases and national organizations endorse their use. The changes made to our departmental processes may not be solely causal. However, national uptake is a gradual process as opposed to the sharp increase in utilization of blocks observed in our study. We did not track patient level efficacy outcomes in our study and thus cannot measure the clinical impact of our program. However, given the literature supporting the efficacy of UGNB procedures in the ED, our program likely improved patient outcomes (16). Additionally, no patient safety events were identified through the QA process. Finally, we did not measure provider sentiment regarding nerve block procedures. The large number of blocks performed over the study period suggests providers felt more supported and experienced fewer barriers to performing UGNBs.

Despite these limitations, our study supports the concept that addressing organizational and provider level barriers in a systematic way can result in a positive change in departmental practices. For departmental leaders aiming to improve care, careful assessment of provider decision making with attention to barriers can serve as a guide. For faculty groups, identifying and supporting a faculty champion who is determined and passionate is vital. The value of trainee driven cultural change is also important to consider, as it was central to progress in our study.

Future research should focus on the benefit of programs such as ours on patient outcomes. In particular, our nerve block program has the potential to impact not only individual patient outcomes but the performance of the hospital system generally. An effective nerve block program has the potential to reduce unnecessary hospital admissions, decrease hospital length of stay for admitted patients, and reduce the complications of hospitalization particularly for vulnerable populations like the older adults. Future analyses will address impact on effectiveness outcomes (e.g., safe opioid prescribing, pain management scores) to demonstrate the wide-ranging benefits of effective pain control in the ED.

## 5 Conclusion

The implementation of a comprehensive ultrasound-guided nerve block program in the University of Colorado Hospital Emergency Department demonstrates how deliberate, incremental system redesign can overcome long-standing barriers to adoption. Through the application of the PRISM framework, sustained leadership engagement, resident and faculty champions, interdepartmental collaboration, and integration of procedural, educational, and operational supports, our department achieved a substantial and sustained increase in UGNB utilization where previously none existed. This initiative highlights the effectiveness of combining top-down structural changes with bottom-up cultural and educational efforts to drive procedural innovation in the ED. Importantly, these gains were achieved without reported safety events, underscoring the feasibility and safety of ED-based UGNB programs. Our experience provides a reproducible model for other institutions seeking to enhance multimodal pain management, reduce opioid reliance, and build sustainable, patient-centered procedural programs within emergency care.

## Data Availability

The original contributions presented in the study are included in the article/supplementary material, further inquiries can be directed to the corresponding author.
